# Efficient estimation of the maximum metabolic productivity of batch systems

**DOI:** 10.1186/s13068-017-0709-0

**Published:** 2017-01-31

**Authors:** Peter C. St. John, Michael F. Crowley, Yannick J. Bomble

**Affiliations:** Biosciences Center, National Renewable Energy Laboratory, 15013 Denver W Pkwy, Golden, CO 80401 USA

**Keywords:** Flux balance analysis, Dynamic optimizations, Elementary flux modes, *Actinobacillus succinogenes*, *Escherichia coli*

## Abstract

**Background:**

Production of chemicals from engineered organisms in a batch culture involves an inherent trade-off between productivity, yield, and titer. Existing strategies for strain design typically focus on designing mutations that achieve the highest yield possible while maintaining growth viability. While these methods are computationally tractable, an optimum productivity could be achieved by a dynamic strategy in which the intracellular division of resources is permitted to change with time. New methods for the design and implementation of dynamic microbial processes, both computational and experimental, have therefore been explored to maximize productivity. However, solving for the optimal metabolic behavior under the assumption that all fluxes in the cell are free to vary is a challenging numerical task. Previous studies have therefore typically focused on simpler strategies that are more feasible to implement in practice, such as the time-dependent control of a single flux or control variable.

**Results:**

This work presents an efficient method for the calculation of a maximum theoretical productivity of a batch culture system using a dynamic optimization framework. The proposed method follows traditional assumptions of dynamic flux balance analysis: first, that internal metabolite fluxes are governed by a pseudo-steady state, and secondly that external metabolite fluxes are dynamically bounded. The optimization is achieved via collocation on finite elements, and accounts explicitly for an arbitrary number of flux changes. The method can be further extended to calculate the complete Pareto surface of productivity as a function of yield. We apply this method to succinate production in two engineered microbial hosts, *Escherichia coli* and *Actinobacillus succinogenes*, and demonstrate that maximum productivities can be more than doubled under dynamic control regimes.

**Conclusions:**

The maximum theoretical yield is a measure that is well established in the metabolic engineering literature and whose use helps guide strain and pathway selection. We present a robust, efficient method to calculate the maximum theoretical productivity: a metric that will similarly help guide and evaluate the development of dynamic microbial bioconversions. Our results demonstrate that nearly optimal yields and productivities can be achieved with only two discrete flux stages, indicating that near-theoretical productivities might be achievable in practice.

## Background

Microbial bioconversion plays a critical role in efforts to enable sustainable production of commodity chemicals from renewable feedstocks. The economic feasibility of the integrated biorefinery concept therefore hinges sharply on the productivity, yield, and titers that can be achieved by a given microbial host [1]. Flux balance modeling has emerged as an important tool in guiding experimental efforts in strain design by predicting the effects of gene knockouts and overexpression on metabolite yields [2]. In developing engineered organisms for optimal performance in batch cultures, a trade-off is often encountered between the productivity and yield that can be obtained via metabolic interventions [3]. As a result, many existing strategies for strain design such as OptKnock or OptForce involve designing mutations to achieve the highest possible yield while maintaining growth viability [4, 5]. Other strategies, including DySScO, have specifically addressed the importance of productivity through dynamic simulations [6, 7]. Overall, approaches tend to follow the principle of designing static networks with minimum metabolic functionality to achieve desired product yields [8]. While these methods are computationally and experimentally tractable, optimum productivity is likely achieved by a dynamic strategy, in which the partition of resources between biomass and product formation varies with time [9].

Experimental studies have investigated the use of multi-stage fermentations to increase productivity and/or yield. These techniques range from simple manipulations, such as changing from aerobic to anaerobic conditions [10, 11], to more complex genetic toggle switches [12] or otherwise inducible gene expression [13]. Computational methods have also been developed to aid in optimizing two-stage fermentation systems. Gadkar et al. optimized the flux profile of glycerol kinase to maximize the productivity of glycerol from glucose [14]. Similarly, Anesiadis and coworkers proposed an extension to Gadkar’s work, where parameters for a quorum-sensing toggle switch are tuned to provide the optimal repression for a target gene to maximize ethanol productivity [15]. By choosing a single reaction target as their control strategy and ensuring that unmodulated fluxes are constrained to maximize a growth objective, these strategies follow similar, well-known techniques such as MOMA [16] or ROOM [17] in calculating an expected biological response from an experimental perturbation. However, a method to calculate a theoretically optimal global limit for a batch culture’s metabolic productivity does not currently exist.

The calculation of maximum theoretical yield is well established in the metabolic engineering literature, and its use helps guide strain design and pathway selection for static knockout and/or overexpression efforts. Maximum yields are useful even though they are not physically realizable: they reveal the cofactor balancing and pathway split ratios necessary to achieve optimum carbon conservation. In an analogous fashion, an estimate of the maximum theoretical productivity of a batch culture system would prove equally useful for the growing field of designing dynamic metabolic interventions. However, due to the computational burden associated with optimizing over all feasible flux profiles, no generalized framework for such an estimate currently exists.

In this article, we present an efficient method for calculating the maximum theoretical productivity of a batch system based on dynamic optimization [18]. Dynamic optimization is a mathematical approach for solving problems involving a differential equation for a desired endpoint objective, in this case the maximum product production in the minimum time. Rather than guessing values for the metabolic state and repeatedly integrating the system forward in time, the approach involves dividing the time domain into a set of finite elements, and within each, representing the dynamic system by a collection of interpolating polynomials. The polynomials are constrained to be continuous between each finite element, and at each of a set of defined points, constraints are imposed to ensure the derivatives of the interpolating polynomials are consistent with maximum substrate uptake rates and maintenance requirements. In this manner, optimal metabolite profiles are found in a single optimization using a large-scale nonlinear programming solver. Dynamic optimization has previously been used in metabolic modeling, including in dynamic flux balance analysis (DFBA) [19] and in calculating optimal control for fed-batch fermentations [20]. However, its widespread usage has been limited by the number of variables that can be simultaneously considered. We therefore remove the explicit mass balance constraints by calculating elementary flux modes [21], and reduce the dimensionality of the optimization problem using yield analysis [22]. Our method accounts explicitly for an arbitrary number of fermentation stages, and allows metabolic fluxes to change continuously over the course of the entire simulation. The method therefore prioritizes finding reasonable upper bounds for the productivities and yields of a given bioprocess over the faithful representation of a cell’s response to genetic perturbation. Since productivity and yield cannot be simultaneously maximized, we demonstrate how this method can be easily extended to calculate the complete productivity vs. yield Pareto frontier. We apply our method to succinic acid production in two microbial hosts: engineered *Escherichia coli* and native *Actinobacillus succinogenes*. Succinic acid is a precursor to commodity chemicals in several industries, and a promising intermediate in the development of sustainable chemical production routes [23]. We show that the method can be useful in strain choice by comparing optimal productivity–yield surfaces for two organisms, and further demonstrate that nearly optimal yields and productivities can be achieved with only two discrete flux stages.

## Methods

### Dynamic flux balance analysis

In flux balance analysis (FBA) models, intracellular metabolic reactions are represented by a stoichiometric matrix $\mathbf {S}$, such that $S_{ij}$ represents the quantity of metabolite *j* produced (or consumed) by reaction *i*. Fluxes through each reaction are represented by the vector $\mathbf {v}$. It is assumed that the time scales associated with intracellular metabolite equilibria are much faster than those associated with cell growth or changes to external metabolite concentrations, and therefore that metabolites in the cell can be modeled using a pseudo-steady-state approximation, $\mathbf {Sv} \approx \mathbf {0}$.

FBA models can be extended to consider the dynamics of substrate consumption and biomass formation by including specific bounds on exchange fluxes and allowing the accumulation or depletion of external metabolites. The dynamic system for cell growth considered in this paper is

1 $$\begin{aligned} \frac{\text{d}x_i(t)}{\text{d}t} = v_i (t) \; x_0(t) \quad \mathrm {for}\; i \in [0, N_X], \end{aligned}$$

where $\mathbf {x}(t)$ represents the concentration of external boundary species, chosen such that $x_0(t)$ is the concentration of dry cell weight (DCW, in grams), and $\mathbf {v}(t)$ represents the flux through each reaction in the cell (in $\mathrm {mmol}\; \mathrm {g}_{\mathrm {DCW}}^{-1}\; \mathrm {h}^{-1}$), chosen such that $v_0(t)$ through $v_N$ represent the exchange flux for species $x_0$ through $x_\text{N}$, respectively. The flux through the first reaction is therefore the specific growth rate ($v_0(t) \equiv \mu (t)$), and is in units of h^−1^. The resulting dynamic flux balance analysis (DFBA) model takes the form of an ordinary differential equation with an embedded linear program, for which efficient methods for the solution of the initial value problem have been developed [24]. However, unlike in dynamic flux balance analysis, in this study we do not impose the optimality of a given cellular objective at each moment in time.

The objective considered in this paper is to maximize productivity of the desired metabolite, $x_\text{p}$, by finding the optimum intracellular flux profiles $\mathbf {v}(t)$ and final fermentation time $t_\text{f}$, subject to steady-state constraints, reaction reversibility, and substrate uptake:

2 $$\begin{aligned} \max _{\mathbf {v}(t), t_\text{f}} {}\quad\frac{x_\text{p}(t_\text{f}) - x_\text{p}(t_0)}{t_\text{f}} \\ \mathrm {such \ that}\quad {}\mathbf {S} \mathbf {v}(t) = 0, \\ \quad{}\mathbf {v_{lb}}(t) \le \mathbf {v}(t) \le \mathbf {v_{ub}}(t).\\ \end{aligned}$$

The optimization in Eqs. 1 and 2 therefore takes the form of an optimal control problem, which can be solved by discretizing in time and solving the problem using dynamic optimization. In previous applications of dynamic optimization in DFBA models, intercellular fluxes are either optimized directly [20] or replaced with representative input–output reactions found via pathway analysis [19]. As each dynamic variable requires a number of parameters to fully specify its shape over the course of the fermentation, minimizing the number of modeled fluxes helps ensure the optimizations converge. In this study, we take advantage of the productivity objective to select Pareto-optimal pathways, greatly reducing the dimensionality of the optimization.

### Calculation of elementary flux modes

To select the optimal metabolic pathways, we calculate elementary flux modes (EFMs) for both of the considered metabolic networks. An EFM is a vector $\mathbf {r}$ in the right nullspace of $\mathbf {S}$ ($\mathbf {S}\mathbf {r} \equiv 0$), such that no other elementary mode has nonzero entries that are a subset of the nonzero entries of $r_i$ [21, 25]. EFMs contain the important property that any vector in the right nullspace of $\mathbf {S}$, i.e., any feasible steady-state flux, can be expressed as a nonnegative combination of the elementary modes $\mathbf {r}$ [26]. Because of this property and because we are only interested in the effect of an elementary mode on the maximum productivity, we can condense the complete set of EFMs to a convex hull in the projection in which we are interested [22]. We further restrict our analysis to the Pareto front of EFMs which allow optimum productivity, removing inefficient modes without affecting the optimal solution.

### Dynamic optimization

We find the flux profiles that achieve the maximum productivity via orthogonal collocation on finite elements, a method for solving endpoint problems involving a dynamic system without an embedded ordinary differential equation (ODE) integrator [18]. In the method, the dynamic system is represented by a series of algebraic constraints that implement an implicit Runge–Kutta method. The resulting sparse nonlinear program is then solved via a large-scale nonlinear programming (NLP) solver. Following the approach of orthogonal collocation [27], we represent the state variables $\mathbf {x}(t)$ from Eq. 1 as a collection of Lagrange interpolating polynomials. A summary of the dimensions and variables used in this method are presented in Tables 1 and 2.

**Table 1:** Dimensions of the NLP problem

Dimension	Index	Description
$N_X$	*i*	Number of state variables
$N_K$	*j*	Number of finite elements
$N_\text{D}$	*k*	Degree of collocation polynomials
$N_\text{F}$	*l*	Number of fermentation stages
$N_R$	*m*	Number of elementary flux modes

**Table 2:** Variable matrices optimized by nonlinear programming

Variable	Shape	Description
$\mathbf {X}$	$(N_X, N_K, N_\text{D})$	Nodes of the Lagrange polynomials interpolating $\mathbf {x}(t)$
$\mathbf {Y}$	$(N_\text{F}, N_R)$	Fractional expression of each elementary mode by stage
$\mathbf {A}$	$(N_K, N_\text{D})$	Total flux at each collocation point
$\mathbf {h}$	$(N_\text{F})$	Length of each of the finite elements within a stage

These matrices are flattened to a single parameter vector prior to being passed to IPOPT

The time domain *t* of the fermentation is divided into $N_K$ finite elements with a nondimensionalized internal time $\tau \in [0, 1]$. Within each finite element, we represent the state vector by a polynomial of degree $N_\text{D}$ at $N_\text{D} + 1$ collocation points, denoted $\tau _n$. We use Gauss–Radau collocation for its stiff decay, and therefore for $N_\text{D} = 5$, we set $\tau _k \in \left\{ 0, 0.06, 0.28, 0.58, 0.86, 1.00\right\}$:

3 $$\begin{aligned} \mathbf {x}_j(\tau ) {}&= \sum\limits_{k=0}^{N_\text{D}}\varvec{\ell }_k(\tau )\mathbf {x}_{jk}\\ \mathrm {where}\quad \varvec{\ell }_k(\tau ) {}&= \prod\limits_{n=0, \; n \ne k}^{N_\text{D}} \frac{\tau - \tau _{n}}{\tau _k - \tau _{n}} \end{aligned}$$

To model changes in the flux distribution, the finite elements are allocated between $N_\text{F}$ distinct fermentation stages. Within each stage, we assume the fractional distribution of elementary modes is held constant, while the total flux through the system is allowed to vary. The dynamic system can thus be calculated by

4 $$\begin{aligned} \frac{\text{d}\mathbf {x}_{ij}}{\text{d}t} = \frac{1}{h_l}\frac{\text{d}\mathbf {x}_{ij}}{\text{d}\tau } = a_{jk} x_{0jk} \mathbf {R} \; \mathbf {y}_l \end{aligned}$$

in which $\mathbf {R}$ is a matrix of shape $(N_R, N_X)$ that contains the chosen elementary modes; $\mathbf {y}_l$ contains the fractional distribution of each elementary mode in the given stage; $a_{jk}$ is the time-varying combined flux through all elementary modes; and $x_{0jk}$ represents the current biomass concentration at the given collocation point. In addition to changes in fractional EFM distribution, the step size $h_l$ is also optimized by the nonlinear program and allowed to vary between fermentation modes.

Solution of the problem involves the constrained optimization over the variables found in Table 2 of the productivity objective

5 $$\begin{aligned} \max _{\mathbf {X}, \mathbf {Y}, \mathbf {A}, \mathbf {h}} \frac{x_{\text{p},N_K}(1) - x_{\text{p},0}(0)}{\sum _{j=0}^{N_K} \mathbf {h}(j)}. \end{aligned}$$

Orthogonal collocation imposes a number of constraints during the optimization process (Eqs. 6, 7, 8, 9, 10). The first of these constraints is that state variable profiles must obey system dynamics, accomplished by ensuring that the derivative of the interpolating polynomial defined in Eq. 3 is equal to the dynamics defined in Eq. 4 at each collocation point. Limits are also placed on the specific uptake and secretion rates at each collocation point in accordance with biological measurements, and the sum of the fractional expression of each elementary mode for the given fermentation stage is constrained to unity.

6 $$\begin{aligned} \mathrm {for} \; &{}j \in [0, N_K];\; k \in [1,N_\text{D}]{:}\\ &{}h_l a_{jk} x_{0jk} \mathbf {R} \; \mathbf {y}_l - \sum\limits_{n=0}^{N_\text{D}}\frac{d\varvec{\ell }_n(\tau _k)}{d\tau } \mathbf {x}_{jn} = 0\\ &{}\mathbf {v_{lb}}(\mathbf {x}_{jk}) \le a_{jk} \mathbf {R} \; \mathbf {y}_l \le \mathbf {v_{ub}}(\mathbf {x}_{jk}) \\ & {}\sum\limits_m^{N_R} y_\text{{lm}} = 1 . \end{aligned}$$

Furthermore, the finite elements are constrained to be continuous:

7 $$\begin{aligned} \mathbf {x}_j(1) - \mathbf {x}_{j+1}(0) = 0 \quad \mathrm {for} \; j \in [0, N_K-1] \end{aligned}.$$

Bounds are also placed on each of the optimization variables:

8 $$\begin{array}{*{20}c} 0 & < & {\mathbf{X}} & < & {1000} \\ 0 & < & {\mathbf{Y}} & < & 1 \\ 0 & < & {\mathbf{A}} & < & \infty \\ {0.1} & < & {\text{h}} & < & {30} \\ \end{array}$$

Finally, two additional constraints are imposed in order to avoid numerical instability and trivial solutions. The relative change in step size between fermentation modes is constrained to a factor of 10,

9 $$\begin{aligned} \frac{\mathbf {h}_{\max }}{\mathbf {h}_{\min }} \le 10, \end{aligned}$$

and the fermentation must consume at least $80\%$ of the initial glucose:

10 $$\begin{aligned} \frac{x^\text{f}_\text{{glc}}}{x^0_\text{{glc}}} \le 0.2 \end{aligned}.$$

### Implementation

Metabolic models are specified using cobrapy [28]. EFMs for each metabolic model are calculated via efmtool [26]. All calculations were performed in Python, using the CasADi library [29] to implement the orthogonal collocation method. IPOPT [30] was used to solve the resulting NLP problem.

## Results and discussion

**Fig. 1:**
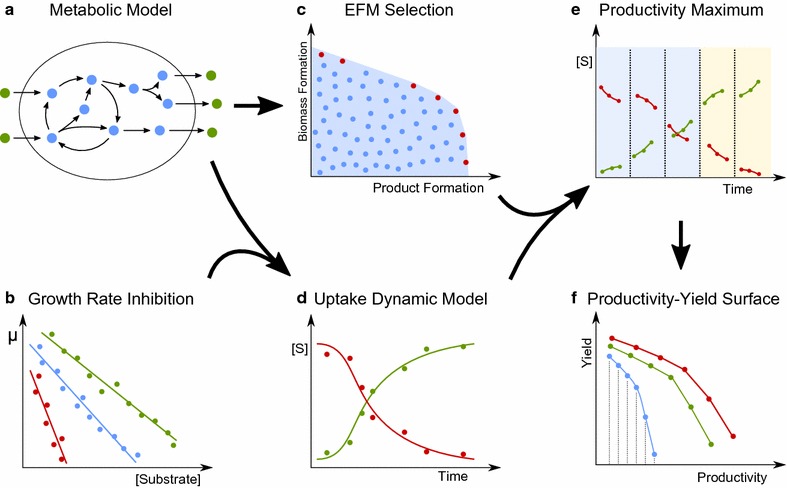
Method overview. A flow chart for how productivity–yield surfaces are calculated. Stoichiometric metabolic models (**a**) are generated from literature sources or genome annotations and are used to calculate the extreme elementary modes of the system (**c**). Literature data from growth experiments (**b**) are used together with stoichiometric models to fit dynamic expressions for substrate uptake kinetics and cellular growth (**d**). Bounds on substrate uptake are used with the set of EFMs in dynamic optimization (**e**) in order to calculate optimal flux profiles. This procedure is repeated to maximize product yield for a number of specified productivity constraints (**e**), generating the surface of maximum yield as a function of desired productivity, (**f**)

We demonstrate the usefulness of the method by calculating the maximum theoretical productivities of succinic acid from glucose for two organisms, *E. coli* and *A. succinogenes*. In a developing a DFBA model, we integrate knowledge of the core-carbon metabolic pathways present in the organism together with experimental data on biomass production and substrate uptake rates. An overview of the computational method is provided in Fig. 1.

### Core-carbon models of *A. succinogenes* and *E. coli*

#### Actinobacillus succinogenes

A stoichiometric model of core-carbon metabolism in *A. succinogenes* was created based on genomic evidence and a previous metabolic reconstruction [31]. The model consists of 72 metabolites and 89 mass- and charge-balanced reactions. A reaction describing *A. succinogenes* cell growth was constructed from a number of experimental measurements. First, a biomass yield was calculated from measured values of the specific growth rate and glucose uptake rate, $0.414\;\mathrm {h}^{-1}$ and $9.5\; \mathrm {mmol}$, respectively [32]. As the models after reduction only map the input–output relationships of glucose to product, the internal details of the biomass function could be approximated without introducing significant error into the method. The stoichiometry of precursor metabolites for biomass synthesis was therefore adapted from an *E. coli* core-carbon model [33], and scaled to match the known cell composition [34]. Energetic requirements of biomass synthesis, including ATP, NADH, and NADPH demand, were adapted from literature values to match the desired biomass yield [35]. A nongrowth-assisted maintenance value of $4.7\;\mathrm {mmol}\; \mathrm {g}_{\mathrm {DCW}}^{-1} \mathrm {h}^{-1}$ was also enforced, which was determined from the value estimated by Lin and coworkers [36] by finding the amount of ATP that can be produced from 0.308 g of glucose. Dynamic constraints on substrate uptake were also imposed. Substrate and product inhibition on growth rate in *A. succinogenes* have previously been quantified with a Han–Levenspiel model [36]. Using the calculated biomass yield of $0.044\; \mathrm {g}_{\mathrm {DCW}}$mmol^−1^ glucose^−1^, the experimentally determined parameters were converted to constraints on glucose uptake:

11 $$\begin{aligned} v_{\text{glc}, \min } = \frac{v_{\max }\;x_{\text{glc}}}{x_{\text{glc}} + K_\text{s}}\;\prod _n \left( 1 - \frac{x_n}{C_{n}^\star }\right) ^{a_n} \end{aligned}.$$

The parameters used in Eq. 11 are presented in Table 3. Additionally, the lower bound of the flux through nongrowth-associated ATP maintenance was constrained to the estimated value:

$$\begin{aligned} v_{\text{atp}, \min } = 4.7 \end{aligned}.$$

No oxygen uptake was allowed to simulate anaerobic growth.

**Table 3:** Parameters for the maximum specific glucose uptake, adapted from values estimated by Lin et al. [36]

Parameter	Value	Units
$v_{\max }$	$-11.47$	$\mathrm {mmol}\; \mathrm {g}_{\mathrm {DCW}}^{-1}$
$K_\text{s}$	11.27	mM
$C^\star _{\mathrm {glucose}}$	860.4	mM
$C^\star _{\mathrm {succinate}}$	385.7	mM
$C^\star _{\mathrm {formate}}$	235.3	mM
$C^\star _{\mathrm {acetate}}$	538.8	mM
$a_{\mathrm {glucose}}$	0.603	–
$a_{\mathrm {succinate}}$	1	–
$a_{\mathrm {formate}}$	1	–
$a_{\mathrm {acetate}}$	1	–

#### Escherichia coli

A core-carbon model for *E. coli* central metabolism was taken from Orth et al. [33], which consists of 72 metabolites and 94 reactions. Dynamic models for glucose uptake in *E. coli* typically assume Michaelis–Menten kinetics, in which high concentrations of glucose ultimately saturate the import mechanisms at their maximum value [37–39]. However, this assumption leads to the erroneous result that maximum productivity is achieved at an infinite initial glucose concentration. Since no suitable literature model for substrate-level growth inhibition from the considered substrates could be found, glucose uptake kinetics were adapted from those used for *A. succinogenes*. Additional bounds on ATP maintenance and oxygen uptake were adapted from those determined by Feist and coworkers [40]:

$$\begin{aligned} v_{\text{atp}, \min } {}&= 8.39\\ v_{o2, \min } {}&= -18.2 . \end{aligned}$$

### Calculation and selection of elementary models for *A. succinogenes* and *E. coli*

Elementary flux modes were calculated to reduce the dimensionality of the optimization and to alleviate the necessity of enforcing stoichiometric equilibrium constraints during the dynamic optimization. For *A. succinogenes*, 4763 EFMs were calculated for growth on glucose and normalized by the glucose uptake rate. After reducing EFMs to the considered boundary species, duplicate EFMs were removed. The boundary species considered for *A. succinogenes* included biomass, ATP, glucose, succinate, formate, acetate, and pyruvate. Since any feasible flux state can be expressed as a nonnegative combination of elementary flux modes, the flux space in the reduced dimensionality can be spanned without loss of generality by only those EFMs at the vertices of a convex hull. Furthermore, as optimal succinate productivity will be achieved using flux modes on the Pareto front of cell growth, ATP production, and succinate secretion, the reduced set of EFMs were further reduced to a final set of 22 Pareto-optimal modes. As EFMs are normalized by glucose consumption, glucose need not be explicitly included in the Pareto surface. For *E. coli*, the additional aerobic growth modes led to a total of 100,273 EFMs. The boundary species for *E. coli* were the same as those used for *A. succinogenes*, with the addition of oxygen and the omission of pyruvate, which was not found to be present in any optimal growth mode. In addition to biomass, ATP, and succinate production, the convex hull of optimal modes in *E. coli* must also consider oxygen consumption. After reduction, 137 modes were kept for *E. coli*.

**Fig. 2:**
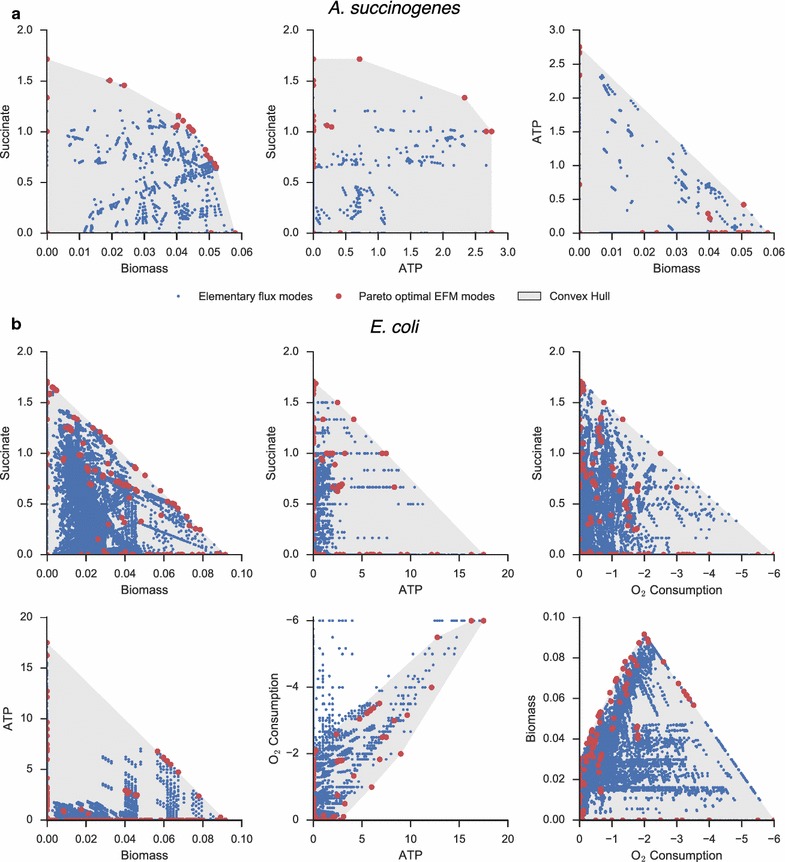
Elementary Flux Mode Surfaces. Projections of the yield surfaces are shown for the 3D yield surface of *A. succinogenes* (**a**) and 4D surface of *E. coli* (**b**). Elementary flux modes are normalized by glucose uptake, and therefore points in yield space represent the amount of product (in mols) which can be produced from 1 mol of glucose. Modes which lie along the Pareto-optimal surface are highlighted in *red*, and the convex hull spanned by these points is* shaded gray*. For *A. succinogenes*, in which succinate production is coupled to optimal growth, the Pareto frontier of succinate vs. biomass yield is sharply curved. Compared with the corresponding plot in *E. coli*, this shape suggests that higher succinate production can be achieved in *A. succinogenes* without a linear penalty in growth and ATP yield. The entire flux cone need not be spanned by the selected EFMs, as demonstrated by the lack of high oxygen consuming—low ATP-producing EFMs selected for the *E. coli* network

The selected EFMs for each organism are shown in Fig. 2, which plots 2D slices of the 3D (A) and 4D (B) yield surfaces. In *A. succinogenes*, the chosen elementary modes can span the entirety of the yield space for the considered boundary species. In *E. coli*, modes that consume high amounts of oxygen while yielding low amounts of ATP are omitted by the algorithm, as they are unlikely to be utilized in the optimal productivity solution.

### Orthogonal collocation

**Fig. 3:**
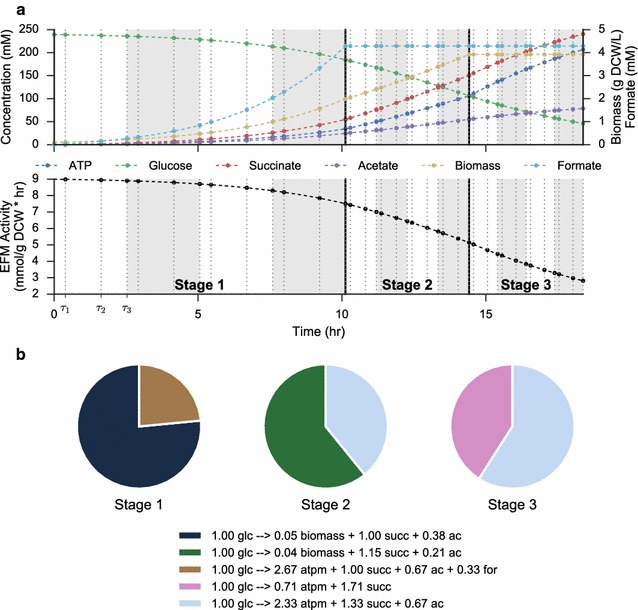
Schematic of the dynamic optimization approach. Example optimum solution for maximum production of succinate in *A. succinogenes*. Time-course trajectories in **a** are broken down into three stages, each of which is composed of a number finite elements (4 in this example, indicated by* light/dark* shading). Within each finite elements, state variables are represented by a lagrange interpolating polynomial at each of 4 collocation points ($\tau _0 \rightarrow \tau _3$), with continuity constraints between finite elements. The step size of the finite elements, *h*, within each stage is also optimized. Metabolic fluxes are optimized by simultaneously optimizing the fractional expression of each elementary mode by stage (**b**) with the overall activity of all elementary modes (**a**,* lower plot*). In this example, high-growth modes are replaced by high succinate yielding modes in later fermentation stages

Optimum productivities are found via a dynamic optimization framework. To further reduce the dimensionality of the optimization, and to allow the effects of discrete fermentation stages to be explicitly simulated, we divide the fermentation time into a number of discrete stages. Within each stage, fluxes are represented by a total flux profile through all elementary modes along with a fractional breakdown of flux through each elementary mode. An example optimum solution for maximum productivity from a 3-stage fermentation in *A. succinogenes* is shown in Fig. 3. For clarity, this example uses only 4 finite elements per fermentation stage, while all other calculations use a minimum of 20 total finite elements evenly divided between stages. The figure demonstrates how the step sizes $\mathbf {h}$ are optimized to achieve the desired dynamic profile. Additionally, since EFMs are scaled by glucose uptake, the activity parameter smoothly tracks the maximum allowable uptake rate. ATP production is enforced at each collocation point by requiring that the flux through the ATP boundary reaction is greater than the nongrowth-associated maintenance requirement. This constraint has the effect of requiring the cells to choose pathways that produce extra ATP for cellular demands, instead of funneling all carbon towards biomass or product production. The selected EFMs in Fig. 3b demonstrate that optimal productivity is achieved by prioritizing cell growth early in the fermentation and succinate production in later stages. The elementary mode that represents the maximum theoretical yield for succinate on glucose, 1.71 mol succinate per mol glucose, is used only partially in the last fermentation stage, highlighting that constraints on cell maintenance prohibit the system from achieving the maximum theoretical yield.

### Effect of increasing the number of fermentation stages

**Fig. 4:**
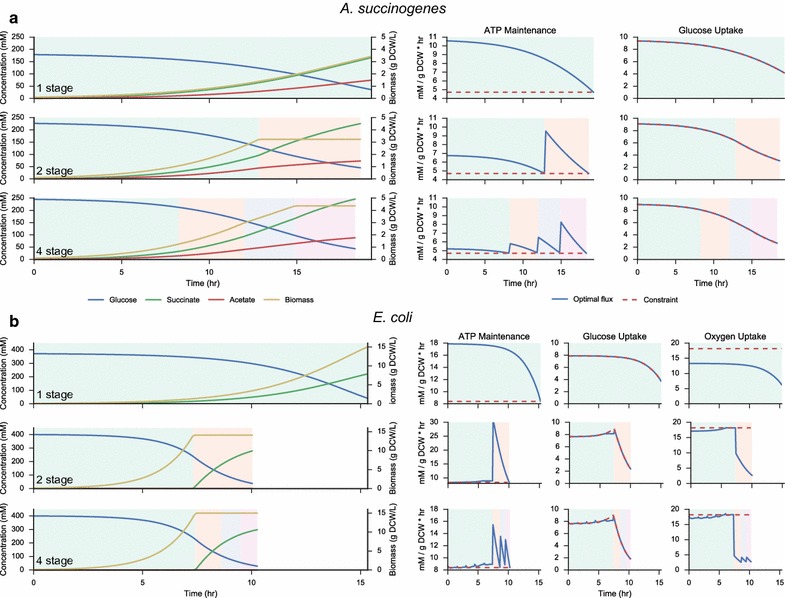
Optimal productivity traces for different numbers of fermentation stages. **a** In *A. succinogenes*, additional stages achieve higher titers while only slightly reducing the optimal fermentation time. This result is achieved by prioritizing growth early in the fermentation and succinate production during the later stages. Additionally, the greater flexibility afforded by more stages allows ATP production to be tailored to meet cellular demand, and less ATP is wasted.** b** In *E. coli*, additional stages drastically reduce the optimum fermentation time as aerobic growth modes are used to increase biomass early in the fermentation. Additional stages beyond two do not drastically change the optimum results, indicating that a simple change from aerobic growth to microaerobic succinate production is close to the global optimum production strategy

In addition to reducing the dimensionality of the problem, splitting the fermentation time into discrete stages with independent EFM expression allows a systematic investigation into the effect of stage count on maximum theoretical productivity. Relative flux ratios within each stage are fixed, and therefore represent consistent enzyme expression. Optimal productivities achieved for a varying number of fermentation stages are shown in Fig. 4 for *A. succinogenes* and *E. coli*. The right-hand side of Fig. 4 plots the dynamic constraints imposed on the solution, including ATP maintenance production rate, maximum glucose uptake rate, and the oxygen uptake rate (for *E. coli*). These constraints are normalized to specific uptake or production rates, and therefore ATP and oxygen uptake constraints remain constant over the fermentation, while the glucose uptake varies based on external concentrations. In all cases, the solutions closely track the maximum allowable glucose uptake rate. In *A. succinogenes*, the transition from one to two stages allows the cell to divide its succinate production strategy into separate growth and product formation stages. The addition of subsequent stages permits slightly higher productivities by the gradual transition from growth to product formation. Additionally, as the flux ratios are fixed within each fermentation stage, the fraction of input carbon diverted to ATP production is also fixed. Thus, as lower extracellular glucose concentrations result in lower glucose uptake rates, the cell must dynamically allocate a higher percentage of energy towards meeting its ATP maintenance requirement. Higher numbers of stages therefore allow the cell to more efficiently meet the maintenance constraint, as demonstrated by lower excess ATP produced with additional stages.

In *E. coli*, maximum succinate production using a single stage is achieved microaerobically. With the addition of a second stage, the optimal fermentation time drops appreciably as succinate production is divided into an aerobic growth phase followed by a microaerobic production phase. The addition of further stages beyond two has little effect on either the succinate productivity or the concentration profiles, serving mainly to keep ATP production closer to the constrained minimum.

### Pareto surfaces of yield versus productivity

While separate estimates of maximum theoretical yield and productivity can provide information on the economic feasibility of a bioprocess, in optimized settings one often cannot be increased without decreasing the other. We therefore show how the given method can be easily extended to calculate a full productivity–yield Pareto surface: the envelope in the multi-objective optimization on which productivity cannot be increased without sacrificing yield. The surface is found by first calculating the maximum productivity ($\mathcal {P}_{\max }$, defined by Eq. 5) via the method described previously.

The nonlinear program is then solved again with yield as the objective,

$$\begin{aligned} \max _{\mathbf {X}, \mathbf {Y}, \mathbf {A}, \mathbf {h}} \frac{x_{\text{p},N_K}(1) - x_{\text{p},0}(0)}{x_{\text{g},0}(0) - x_{\text{g},N_K}(1)}, \end{aligned}$$

in which $\mathbf {x}_\text{g}$ represents the glucose concentration, while holding productivity constrained to a fraction of the maximum productivity,

$$\begin{aligned} \frac{x_\text{p}(t_\text{f}) - x_\text{p}(t_0)}{t_\text{f}} - \alpha \mathcal {P}_{\max } = 0 \quad \mathrm {for}\;\alpha \in [0,1]. \end{aligned}$$

Computational efficiency in the repeated optimizations is improved by using IPOPT’s warm solve method, which preserves the optimal solution as a starting guess for the next iteration.

**Fig. 5:**
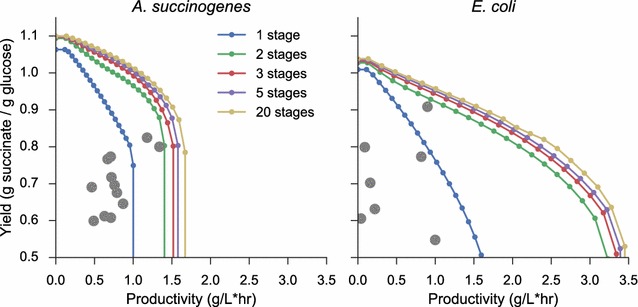
Productivity–yield pareto surfaces for *A. succinogenes* and *E. coli*. Productivity–yield surfaces are calculated by holding productivity constant while maximizing yield for a given number of stages. Experimentally realized productivity and yields are shown in* gray*, with most points falling in the space predicted by single-stage strategies. References for the experimental points are given in Tables 4 and 5 for *A. succinogenes* and *E. coli*, respectively. The results indicate that improving existing yields in *A. succinogenes* fermentations would lead to greater gains than improving productivity. Similarly in *E. coli*, the results show that higher productivities could likely be achieved by leveraging aerobic growth modes more effectively

Figure 5 plots the resulting yield–productivity surfaces for 50 linearly spaced $\alpha$ values. By varying the number of allowed fermentation stages, these surfaces reveal the performance gains which can be achieved by allowing additional metabolic flexibility. For *A. succinogenes*, higher yields can be obtained due to the lower specific ATP maintenance requirement. Additionally, as succinic acid is the naturally predominant fermentation product in *A. succinogenes*, high yields are obtained even at close to the maximum productivity. In *E. coli*, notable performance gains are achieved by moving to a 2-stage fermentation, as it enables efficient usage of aerobic growth modes. In both cases, moving beyond two distinct flux modes does not substantially increase the yields or productivities that can be achieved.

The calculated optimal surfaces are compared to data on succinate yield and productivity that have been achieved experimentally from both wild-type and engineered organisms, compiled in Tables 4 and 5. For consistency with the assumptions of the method, we limit the data to experimental values in batch cultures on grown on glucose without fed-batch operation or biomass recycling. The measured values of biomass yields and glucose uptake kinetics used in modeling vary from experiment to experiment, and thus the estimated productivity–yield surfaces include a degree of uncertainty. However, the data largely fall within the predicted envelope of single-stage processes, confirming the accuracy of the assumptions made in each model. These results illustrate how the proposed method can be useful for guiding experimental effort: as wild-type *A. succinogenes* fermentations naturally fall close to the optimal productivity–yield surface, further genetic manipulation of this organism is unlikely to yield significantly improved performance. Similarly with *E. coli*, improving succinate productivity beyond the values previously recorded will likely require a two-stage process involving aerobic growth and anaerobic succinate production.

**Table 4:** Literature data for productivity and yield achieved in *A. succinogenes* batch fermentations

Productivity (g L^−1^ h^−1^)	Yield (g g^−1^)	Reference
1.18	0.82	[41]
0.71	0.77	[41]
0.49	0.60	[41]
0.67	0.77	[41]
0.76	0.70	[41]
0.63	0.61	[41]
0.46	0.69	[42]
0.72	0.72	[42]
0.71	0.61	[42]
0.87	0.65	[42]
0.55	0.76	[43]
1.34	0.80	[43]
0.66	0.79	[44]
0.75	0.76	[45]

**Table 5:** Literature data for productivity and yield achieved in *E. coli* batch fermentations

Productivity (g L^−1^ h^−1^)	Yield (g g^−1^)	Reference
0.90	0.91	[46]
0.82	0.77	[46]
1.27	0.46	[11]
0.60	0.28	[47]
0.22	0.63	[48]
1.00	0.55	[49]
0.16	0.70	[49]
0.09	0.80	[50]
0.04	0.61	[50]

## Conclusions

This study represents a computationally efficient method for determining the maximum theoretical productivity for a batch culture system. As the fields of metabolic engineering and synthetic biology continue to develop techniques for the dynamic manipulation of metabolism, our methodology will enable experimental efforts to be focused on where the greatest improvements can be expected. While this study has focused on finding globally optimal solutions, future implementations might search for optimal strategies using only experimentally tractable EFM selections (i.e., ones that are growth-optimal for 1 or 2 gene knockouts). The method can also easily be generalized for conversion of multiple substrates as long as appropriate experimental data exist to fit detailed models to substrate uptake kinetics. It could also be extended to explicitly optimize final titer instead or in addition to yield and productivity. Overall, this work emphasizes the need for better empirical models of substrate and product exchange rates and growth kinetics in designing dynamic metabolic interventions.

## Abbreviations

DFBA: dynamic flux balance analysis
FBA: flux balance analysis
EFMs: elementary flux modes
ODE: ordinary differential equation
NLP: nonlinear programming
DCW: dry cell weight
ATP: adenosine triphosphate
NADH: nicotinamide adenine dinucleotide
NADPH: nicotinamide adenine dinucleotide phosphate
